# Who Resembles Whom? Mimetic and Coincidental Look-Alikes among Tropical Reef Fishes

**DOI:** 10.1371/journal.pone.0054939

**Published:** 2013-01-25

**Authors:** D. Ross Robertson

**Affiliations:** Smithsonian Tropical Research Institute, Balboa, Republic of Panamá; University of Kent, United Kingdom

## Abstract

Studies of mimicry among tropical reef-fishes usually give little or no consideration to alternative explanations for behavioral associations between unrelated, look-alike species that benefit the supposed mimic. I propose and assess such an alternative explanation. With mimicry the mimic resembles its model, evolved to do so in response to selection by the mimicry target, and gains evolved benefits from that resemblance. In the alternative, the social-trap hypothesis, a coincidental resemblance of the model to the “mimic” inadvertently attracts the latter to it, and reinforcement of this social trapping by learned benefits leads to the “mimic” regularly associating with the model. I examine three well known cases of supposed aggressive mimicry among reef-fishes in relation to nine predictions from these hypotheses, and assess which hypothesis offers a better explanation for each. One case, involving precise and complex morphological and behavioral resemblance, is strongly consistent with mimicry, one is inconclusive, and one is more consistent with a social-trap based on coincidental, imprecise resemblance. Few cases of supposed interspecific mimicry among tropical reef fishes have been examined in depth, and many such associations may involve social traps arising from generalized, coincidental resemblance. Mimicry may be much less common among these fishes than is generally thought.

## Introduction

Interspecific mimicry involves the evolution of changes in the morphology and behavior of a mimic species that, by increasing its resemblance to another model species, provide benefits to the mimic, such as protection from predators or enhanced access to food (e.g. [1], [2]). The literature on tropical reef fishes contains numerous reports that attribute behavioral associations of look-alike species to mimicry ([2]–[5] for reviews) although few such cases have been investigated in any depth [6]–[13]. In many cases discussion assumes that a behavioral association between two similar species is mimetic, and focuses on basic supporting evidence and plausible potential benefits to the putative mimic. Most studies fail to consider alternative hypotheses that might account for behavioral associations of look-alike species in the absence of mimicry, including associations that involve benefits to the assumed mimic. When they do note alternative explanations those generally are given only passing consideration (but see [14]) and the emphasis is on evidence of support for mimicry.

A sensory trap is a signal that evolved to elicit an out-of-normal-context response by a receiver and from which the signal-producer benefits [15]. Sensory traps are widely involved in mate choice [16], predator–prey interactions, and interspecific mutualisms [17]. Interspecific mimicry is a form of sensory trap in which the evolved signal, the mimic’s model-like appearance, induces a particular response by the mimicry target that benefits the signal producer. The **mimicry hypothesis**, then, proposes that the mimic resembles its model [1] and the mimicry target is the agent of natural selection that produced that resemblance in the mimic. However, an alternative hypothesis can be constructed, based on the “model” and “mimic” independently evolving a common appearance. In this, the **social-trap hypothesis,** a coincidental resemblance of the model to the “mimic” stimulates an out-of-normal-context social attraction of the latter to the model, i.e. the “mimic” is socially trapped by a fish that resembles itself. When an interaction that results from that attraction provides rewards to the “mimic”, then learning reinforces the attraction and leads to the “mimic” regularly associating with the model. In both hypotheses the “mimic” benefits from the relationship, with evolved benefits from mimicry and learned benefits through the social-trap. With a social trap, in contrast to mimicry, the signal (the model’s appearance) did not evolve to produce the out-of-context response (social attraction of the “mimic” to the model), and the signal-producing model gains no benefit from that response, at least in situations that resemble aggressive mimicry (see below). Further, there is no involvement of a selection agent equivalent to a mimicry target in the evolution of the similarity of model and “mimic”. A combination of two existing behaviors by members of its taxon would predispose a “mimic” to develop a social-trap association with a coincidentally similar model: (i) strong social attraction to conspecifics based on appearance cues similar to those displayed by the model, and (ii) the formation of equivalent beneficial associations with dissimilar heterospecifics. Coincidental resemblance of a “mimic” and an unrelated model could arise through independent selection on each for appearance characteristics related to predator avoidance, warning signals, background matching, or intraspecific communication.

Most major types of mimicry have been reported for tropical reef fishes [2], [4], [5], [14], [18], [19]. These include **Aggressive mimicry:** a predator mimics a harmless or beneficial species, and gains enhanced access to prey; **Batesian mimicry:** an prey species gains protection from predators by mimicking a protected (dangerous or beneficial) species; **Mullerian mimicry:** multiple dangerous species evolve a common appearance that reduces predation risks to all; and **Social (or Schooling) mimicry:** a mimic gains protection from predators through its inconspicuousness within schools of an unprotected model. The object of this paper is to stimulate discussion about mimicry among tropical reef fishes in relation to alternatives to the mimicry hypotheses. To do so I examine three well known cases that have been widely interpreted for several decades as aggressive mimicry, one of the commonest types of mimicry thought to occur among reef fishes. Those three were chosen because they incorporate varying degrees of precision in the resemblance of the supposed “mimic” to its model, from very precise and multifaceted to very generalized and simple. The results of this reassessment should reinforce the value of a sense of skepticism in how behavioral associations of look-alike reef fishes are viewed and investigated.

### Methods

I assess each of the three cases in relation to nine predictions relating to the two alternative hypothesis, most of which have been used in previous discussions of aggressive mimicry [1]–[3], [11], [19]. While these predictions vary in importance, and data relating to only a few of them may be sufficient when the resemblance of the mimic to a model is detailed and complex, with imprecise resemblance comprehensive information relating to all nine would strengthen conclusions about whether the relationship is mimetic or based on coincidental similarities in appearance.

#### 1. Quality of resemblance

The closer, the more detailed and the more multifaceted the resemblance of the “mimic” to an unrelated model the more likely is mimicry. Such resemblance features include form (body and fin shapes), size, coloration (color hues and tones+color pattern) and behavior. The more generalized and imprecise the resemblance the greater the possibility that it is coincidental, although mimetic resemblances are not necessarily precise (cf [20]).

#### 2. Geographic variation in resemblance

Geographic variation in the appearance of the model and mimic are linked: variation in the model is mirrored by variation in the mimic, which does not vary independently of the model. Independent geographic variation indicates a coincidental resemblance.

#### 3. Resemblance characteristics of the mimic atypical for its taxon

Resemblance-enhancing characteristics of the “mimic” that are not exhibited by its non-mimic near-relatives support the case for their evolution as mimetic features. Such characteristics include both atypical behavior [2], [21] and atypical morphology (e.g. shape, size and coloration). A lack of such unusual characteristics in the “mimic” is more consistent with coincidental resemblance.

#### 4. Similarity and distinctiveness of model and mimic apparent to target, which can identify status of model

The deception-target is capable of perceiving the model and mimic as similar, but distinct from other species, and of identifying the benign or beneficial status of the model. Inability of the target to do so is consistent only with coincidental resemblance.

#### 5. Relative abundance of model and mimic

The mimic is locally much less common than its model. Such relative abundance also is consistent with coincidental resemblance.

#### 6. Spatial association of model and mimic

There is strong overlap in the distributions of the two species, geographically and in the use of habitats at the same location, and the “mimic” associates with the model. Such association also is consistent with coincidental similarity.

#### 7. Diet overlap of model and mimic

The mimic is a predator that represents a threat to the mimicry target, and its model is harmless or beneficial to the target, a relationship that also is consistent with coincidental resemblance.

#### 8. Evidence of successful deception

The mimicry target demonstrates that it has been deceived by the mimetic resemblance of the “mimic”. Highly successful deception is more indicative of mimicry, weaker deception with coincidental resemblance.

#### 9. Evidence of benefits due to mimicry

There is direct evidence of a benefit to the “mimic” due to its resemblance to the model. Stronger reliance on such benefits is more indicative of mimicry, weaker reliance with coincidental resemblance.

Note: No research permits were required for any of the field observations I made on fishes in connection with this study.

## Results

### I The False-cleanerfish Blenny, *Aspidontus taeniatus,* and the Bluestreak Cleaner Wrasse, *Labroides dimidiatus*

The best known example of mimicry among reef fishes is that of the bluestreak cleaner wrasse *Labroides dimidiatus* by the false-cleanerfish blenny *Aspidontus taeniatus.* The wrasse offers a beneficial service to reef fishes, the removal of ectoparasites, while the blenny bites pieces from the fins of host fishes serviced by the wrasse [1], [3], [6], [22]. *L. dimidiatus* is widespread throughout the Indo-central Pacific. Until recently *A. taeniatus* was considered to have a similar distribution. However, the Indian Ocean form was recently split off as a separate species, *A. tractus*, which looks very similar to *A. taeniatus* [23]. Information below about *A. taeniatus* also relates to *A. tractus*.

#### Quality of resemblance

**Figure 1:**
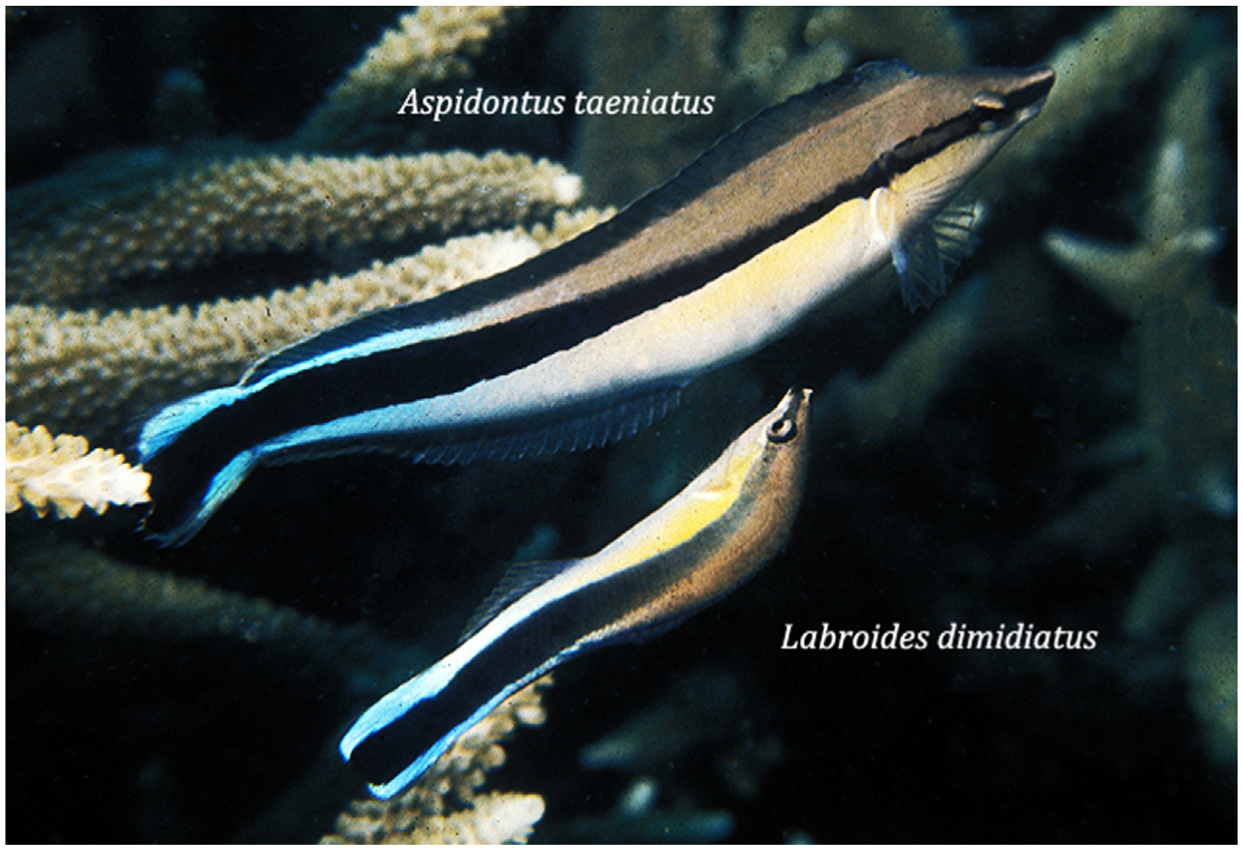
An adult of a mimetic blenny, the false-cleanerfish *Aspidontus taeniatus*, is inspected for cleaning by its model, an adult of the bluestreak cleaner wrasse *Labroides dimidiatus*. Photo: DR Robertson, Great Barrier Reef, 1972.

The resemblance of adult *A. taeniatus* to adult *L. dimidiatus* is very precise, in body size, the shape & proportions of the body and fins, the hues and tones of colors, and the details of color patterning [1], [24] (see Figure 1). Juveniles and adults of *A. taeniatus* have very different coloration, which closely matches the equally different juvenile and adult coloration of *L. dimidiatus* Adult *L. dimidiatus* have very limited capacity to change their coloration (DRR pers obs). In contrast, adult *A. taeniatus* can display at least four different patterns, depending on their motivational state, and the pattern that closely resembles that of *L. dimidiatus* is displayed when the blenny is calmly behaving like the wrasse [1]. Like *L. dimidiatus*, *A. taeniatus* normally uses its pectoral fins for slow swimming, and has the same non-alarmed reaction to close approach by divers and large fishes as the wrasse [3], (DRR pers obs). *L. dimidiatus* perform a highly characteristic “dance” when approaching or inviting the approach of large host fishes, particularly predators, and *A. taeniatus* also simulates this dance [1]. Thus the mimic’s resemblance to its model is of high quality in all major features that clearly identify the beneficial status of the model.

#### Geographic variation in resemblance characteristics

Parallel geographic variation occurs in major parts of the coloration of *A. taeniatus* and *L. dimidiatus* at several locations in the Pacific [5], [25]. Geographic variation in fine details of the color pattern *of L dimidiatus* is also matched by both *A. taeniatus* and *A. tractus* [22] (DRR pers obs on both species). No geographic variation has been noted in the coloration of *A. taeniatus* that is independent of color variation in *L. dimidiatus*, although Russell et al [26] described a color variant of *A. taeniatus* at Samoa that closely resembles an apparently undescribed, co-occurring species of *Labroides*.

#### Resemblance characteristics unusual for the taxon?

Nemophine blenniids include *Aspidontus* and five other genera [25], [27]. There are three species of *Aspidontus*: *A. taeniatus, A. tractus* and *A. dussumieri*. *A. taeniatus* uses labrid-like pectoral sculling for slow swimming. In contrast, members of other nemophine genera use typical blennioid eel-like body-wiggling for slow swimming, and *A. dussumieri* uses an intermediate combination of concurrent pectoral sculling and body-wiggling (W Smith-Vaniz, pers comm. May 2012; J Jensen pers comm. (video), June 2012). *A. taeniatus* has a shorter, more robust body and more pointed snout than both *A. dussumieri* and members of other nemophine genera, which enhances its resemblance to *L. dimidiatus*. Coloration comprised of a pale body plus a dark mid-lateral stripe is not unusual among both nemophines and many other blennies [25], [27]. However, *A. taeniatus* is the only nemophine in which the dark lateral stripe extends onto and broadens across the entire tail fin, as it does in *L. dimidiatus*. *A. taeniatus* and *L. dimidiatus* are sexually monomorphic, unlike *A. dussumieri* which is mildly sexually dimorphic in coloration. Adults of *Aspidontus* have a swim bladder, which facilitates the matching of the *Labroides* swimming mode (labrids have swim bladders) by *A. taeniatus*. However, this is not taxonomically unusual as other, non-mimetic nemophines that routinely swim in mid-water also have swim bladders [27]. Thus there are multiple characteristics of *A. taeniatus* that enhance its resemblance to the cleaner wrasse and that are not seen in its near relatives, notably its body and head shape, a major structural component of its color pattern, and its simulation of various behaviors that identify the beneficial status of the model.

#### Target perceives similarity of model and mimic, and identifies model’s status

Measurements of color hue and luminance of reef fishes by Cheney & Marshall [11] show that coloration of model/mimic pairs likely appear more similar to reef fishes than do coloration of non-mimetic pairs. Two of the mimetic pairs tested in that study included adults of *A. taeniatus* and *L. dimidiatus*, and juveniles of each. Many fish species of greatly varying sizes and trophic and taxonomic groups, including some known to be attacked by *A. taeniatus*, approach *L. dimidiatus* and “pose” (hold a stationary position with fins and mouth spread open) to “invite” and facilitate its cleaning activities [1], [28], (DRR pers obs). As they do not behave in the same way to heterospecific fishes in general these mimicry-target fishes clearly recognize the model’s distinctive beneficial status.

#### Relative abundance of model and mimic

In most localities *A. taeniatus* is much less common than *L. dimidiatus* [1], [3], [6], (DRR pers obs on both *A. taeniatus* and *A. tractus*). However, Kuwamura [6] described *A. taeniatus* as sometimes reaching a considerably greater local abundance than *L. dimidiatus*, which he related to temporary mating activities of the blenny.

#### Association in space of model and mimic

The geographic range *L. dimidiatus* completely encompasses those of *A. taeniatus* and *A. tractus* (see www.iobis.org), and both wrasse and blenny occupy the same shallow reef habitats [3], [6], (DRR pers obs). However, *A. taeniatus* do not commonly closely associate with feeding *L. dimidiatus*. The blenny is attacked by adult cleaners if it intrudes into the small, fixed cleaning stations where most of their cleaning interactions take place (DRR pers obs), and many of the attacks on fishes by *A. taeniatus* occur away from the immediate vicinity of *L. dimidiatus’* cleaning stations [6], (DRR pers obs).

#### Diets of model and mimic

*L. dimidiatus* eats ectoparasites, skin fragments and mucus from host fish body surfaces [28]. *A. taeniatus*, in contrast, eats pieces of fin snipped from other species of fishes, benthic fish eggs plundered from the nests of parent fishes, and pieces of gill filament ripped from benthic tubeworms [6]. The diet of the blenny also appears to vary geographically, and may sometimes include little in the way of fish-fin pieces [6].

#### Evidence of success of mimicry

Fishes of a variety of species that are often cleaned by *L. dimidiatus* approach and “pose” to *A. taeniatus* in the same manner they do to invite cleaning by *L. dimidiatus* [6], (DRR pers obs). Such deceived targets include large predatory fishes that represent a threat to the blenny [6]. Young *L. dimidiatus* occasionally pose to *A. taeniatus* as though inviting cleaning (DRR pers obs), indicating success of the mimicry. However, experienced target fish do learn to discriminate between *A. taeniatus* and *L. dimidiatus* and avoid or attack the blenny [3], [6], [22], (DRR pers obs).

#### Evidence of benefits due to mimicry

There are two lines of evidence of benefits. First, host fishes pose to *A. taeniatus* as they do to *L. dimidiatus*, which facilitates the blenny’s attacks on them. Sometimes the same host individual will pose to and be attacked by the same blenny several times in rapid succession before avoiding the blenny (DRR pers obs). Second, large predators that invite cleaning by *L. dimidiatus*, behave in a similar manner to A. *taeniatus* [6], demonstrating that they do not invariably treat the blenny as they do other potential prey.

#### Mimicry versus coincidental resemblance

In this case a range of aspects of the morphological and behavioral resemblance of the blenny to the wrasse are too precise to be coincidental. Those include various characteristics of the blenny that are unusual for its taxon. All nine predictions of the mimicry hypothesis are clearly supported by observational field data, and that hypothesis accounts for the full combination of aspects of the relationship between *L. dimidiatus* and *A. taeniatus*. None of the data relating to the nine predictions is better explained by social-trapping due to a coincidental resemblance or are inconsistent with mimicry (see Table 1).

**Table 1:** Summary of support for 3 cases of supposed aggressive mimicry.

Mimicry hypothesis: 9 predictions	Three Proposed Mimics		
	*Aspidontus taeniatus*	*Hemiemblemaria simulus*	*Hypoplectrus* (8 species)
Good, detailed resemblance in form (shape, coloration) & behavior	Yes (in form, specialbehaviors)	Yes (in form)	In form: 1–2 species good; others moderate/poor
Parallel geographic- & age-variation in form	Yes: geographic & agein coloration	No geographic variation;juvenile “mimic” unlikejuvenile “model”	Geographic variation non-parallel; juvenile “mimics” unlike juvenile “models”
Taxonomically unusual resemblance features	Yes: various, in form& behavior	Yes: in form (butsignificance equivocal)	None known
Target can perceive model & mimic as similarand identify benign status of model	Perception: yes (form,color & behavior) Modelstatus: yes	Perception; yes (form & color,if target is fish). But “model” &“mimic” both non-benign	Perception & identification: fish targets – yes; crustacean targets – no? (visually incompetent?)
Abundance: model>mimic	Yes	Yes	Yes 6 cases; no 2 cases
Close spatial association of model & mimic	Yes	Yes	Yes: all cases
No model-mimic diet overlap	True	Quantitative difference only	True: all cases
Evidence of successful deception	Yes for multiple targets	None	Perhaps 1 species; rest no
Evidence of benefits due to deception	Yes for multiple targets	None	Perhaps 1 species; rest no
**Support for mimicry vs coincidental resemblance**	Strong	Equivocal	Coincidental resemblancemore likely
**Mimicry is as originally proposed?**	Broader	Different, if present	If present

Reef fish species vary in their ability to discriminate between coloration of different fishes, have poorer visual acuity than many other vertebrates and may be less able to discriminate between differences in fish coloration than are humans [11], [29], [30]. Why does the similarity of *A. taeniatus* to *L. dimidiatus* seem so precise to the human observer? Several factors may contribute here. Interactions between *A. taeniatus* and the targets it attacks often occur at close range and involve active cooperation of the victims, and highly detailed resemblance may slow such targets’ learning to discriminate between model and mimic. Further, behaving like the cleaner wrasse makes the blenny particularly vulnerable to predator attacks and a detailed resemblance may reduce such a risk. In addition, Wicker [1] also noted that (i) the *A. taeniatus* mimicry is aimed at a range of taxa of targets that have differing visual capabilities, and (ii) host fishes learn the identity of the geographically variable cleaner as well as its mimic, and learned characteristics tend to be more finely tuned than innately recognized characteristics. By being able to learn the identity of the blenny and avoid or attack it, experienced hosts clearly demonstrate they have the necessary visual capabilities to distinguish minor differences between model and mimic, indicating the value of a precise resemblance.

The major question arising from existing work on *A. taeniatus* concerns the nature of the mimetic relationship. Most published accounts treat it as a simple aggressive mimicry that facilitates the blenny’s fin-clipping of cleaner-fish hosts. However, based on the only detailed study of the ecology of *A. taeniatus*, Kuwamura [6] proposed that major mimicry targets are predatory fishes capable of eating *A. taeniatus* (see also [1]), ie. that the mimicry is primarily Batesian. Further work on *A. taeniatus* and *A. tractus* would be useful to clarify the nature and extent of variation in the mimetic relationships across its geographic range.

#### Conclusion

There is strong support for the mimicry hypothesis in this case, and the data are consistent with all nine predictions of this hypothesis (Table 1). However, the mimicry probably is broader (Aggressive plus Batesian) and more variable geographically than originally thought.

### II The Wrasse-blenny, *Hemiemblemaria simulus*, and the Bluehead Wrasse, *Thalassoma bifasciatum*

The resemblance of the wrasse blenny, *Hemiemblemaria simulus*, to Initial Phase (IP) individuals of the biphasic bluehead wrasse, *Thalassoma bifasciatum*, was labeled as mimetic by Longley and Hildebrand [31] in their species description of the blenny. Randall and Randall [3] proposed that the blenny may be both a Batesian mimic because the wrasse is a “protected” species that cleans parasites from other fishes, and an aggressive mimic that gains enhanced access to small prey fishes that do not represent wrasse prey.

#### Quality of the resemblance

**Figure 2:**
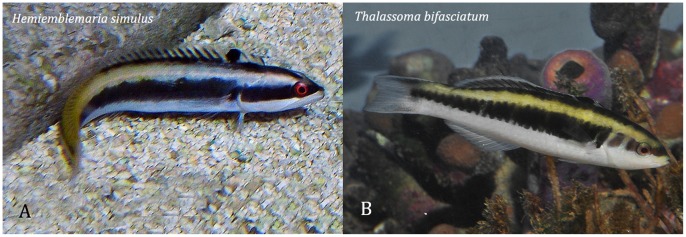
The wrasse blenny *Hemiemblemaria simulus,* and its supposed model, the bluehead wrasse, *Thalassoma bifasciatum.* Photos: A - J Adams; B - DR Robertson.

To the human observer the visual resemblance of *H. simulus* to IP *T bifasciatum* is good in several aspects [3], [31]-[33] (see Figure 2). Adults of *H. simulus* have similar size, and body and fin shapes to IP blueheads, the smallest and most abundant color phase of the wrasse, and a similar pectoral-sculling swimming mode to the wrasse [31]. Adults of the blenny and IP blueheads both vary in color, and there are similarities in three quite distinct color patterns seen in preserved specimens of both species [3]. However, those three patterns represent only part of the IP blueheads’ live-color repertoire (DRR pers obs). Blennies generally have strong ability to change between quite different color patterns, and the circumstances under which *H. simulus* displays different color patterns, whether an individual can change between those patterns, and how their display that might relate to the color patterning of IP blueheads with which the blenny is associating at any time are not known. In contrast to the adult, the different color pattern of juvenile *H. simulus* does not resemble that of juvenile blueheads [32], [33]), which look like miniature IP fish.

#### Geographic variation in the resemblance

No geographic variation in the coloration of either species has been described. However, very little work has been done with the blenny.

#### Resemblance characteristics unusual for the taxon?

The general morphology of *H. simulus* is unlike that of any other chaenopsid blenny: a short, stubby, semi-fusiform body, and a long, pointed, compressed head virtually devoid of cirri [34]. In contrast, members of its sister taxon, *Ekemblemaria* [35], have a much more elongate body, and a short blunt head with large branched cirri. *Ekemblemaria* species have dark colors with blotches and vertical bars rather than a longitudinal stripe like *Hemiemblemaria* (and IP blueheads). Both genera are part of the *Chaenopsis* clade, members of which tend to be more free-swimming, often by using pectoral sculling, than other chaenopsids [35]. Pectoral-swimming is taken to extremes in *Hemiemblemaria* (and *Lucayablennius*, another chaenopsine that often swims in midwater). Other chaenopsids have the typical blennioid swimming mode: eel-like wiggling of the body. *H. simulus* and the monotypic *Lucayablennius* have some of the most divergent color patterns of any chaenopsids.

Unusually for blenniids, *Aspidontus* and its near relatives that swim in midwater have swim bladders [27], a feature that facilitates mimicry of its model’s swimming mode by *A taeniatus* (see above). At my request W Smith-Vaniz dissected museum specimens of various chaenopsids and found (WS-V pers com, May 2012) a well developed swim bladder in *Hemiemblemaria,* but not in *Ekemblemaria* (1 species), *Chaenopsis* (1 species), *Lucayablennius* (1 species), *Acanthemblemaria* (2 species), *Emblemaria* (1 species), or *Protemblemaria* (1 species). *Hemiemblemaria* appears to have evolved a unique (for chaenopsids) capacity that facilitates mid-water living and pectoral-swimming, and which enhances its behavioral resemblance to IP blueheads. Thus *H. simulus* has a suite of morphological and behavioral features that are unusual for its family, some of which are evident in a less developed form among other members of its clade, and many of which enhance its resemblance to IP blueheads.

#### Target perceives similarity of model and mimic, and identifies model’s benign status

Although Cheney and Marshall [11] did not test the similarity in coloration of adult *H. simulus* and IP *T. bifasciatum*, they did compare mimetic pairs of fishes with similar coloration (black stripes on a yellow and white background). Their results indicate that shallow water Caribbean reef fishes likely perceive the coloration of *H. simulus* adults and IP blueheads as similar. However, as all prey types consumed by the blenny are also eaten by the bluehead (see below) IP blueheads would not be perceived as benign by the blenny’s prey, although the risk of attack from each may differ.

#### Relative Abundance of “model” and “mimic”

*H. simulus* is far rarer than the abundant bluehead wrasse, which is one of the commonest fish on Caribbean area reefs [3], [31], (DRR pers obs).

#### Spatial association of “model” and “mimic”

The geographic range of *T. bifasciatum* entirely encompasses that of *H. simulus* [36], and the blenny lives in shallow areas occupied by the ubiquitous wrasse [31]. Individual *H. simulus* join feeding aggregations of IP blueheads, but also associate with at least one other species of wrasse that adults of the blenny do not resemble [30].

#### Diets of model and mimic

*H. simulus* eats mainly small free-swimming crustaceans, plus substantial numbers of small fish [3]. The bluehead eats small mobile benthic invertebrates, demersal fish eggs, free-swimming crustaceans, small numbers of ectoparasitic crustaceans from the skins of fishes, and small fishes [37]). Given the opportunity, IP blueheads readily attack and eat small fish of a size eaten by *H. simulus* (DRR pers obs). Thus the bluehead eats the same food types as the wrasse-blenny, although those represent different proportions of the diets of blenny and wrasse.

#### Evidence of success of mimicry

Given that IP blueheads represent predators of blenny prey it is unclear how a resemblance to blueheads might enhance the blenny’s predation, and there is no evidence that such happens.

#### Evidence of benefits due to mimicry

There is no direct evidence of aggressive-mimicry benefits to the blenny arising from its resemblance to IP blueheads.

#### Mimicry vs coincidental resemblance

Several lines of evidence support the mimicry hypothesis in this case: the strong resemblance of adult *H. simulus* to IP blueheads in a combination of general morphology, coloration and swimming behavior, characteristics that are atypical for the blenny’s taxon, strongly atypical in the case of shape, coloration and pectoral swimming facilitated by a swim bladder. The blenny is much less common than the wrasse, there is good spatial overlap between blenny and wrasse, and the blenny does sometimes closely associate with the wrasse.

However, important evidence is inconsistent with aggressive mimicry in this case. First, IP blueheads eat all of the same food types as the blenny, albeit in smaller proportions. Thus the wrasse represents a threat to all the blenny’s prey rather than a benign model for an aggressive mimicry. Second, much of the cleaning of other fishes that IP blueheads do is done by juvenile blueheads, which clean relatively large host fishes [38], (DRR pers obs), ie fishes that do not represent *Hemiemblemaria* prey. Juvenile blueheads may enjoy some “beneficial-status” protection from predation, at least within the context of their cleaning activities. However, such protection does not extend to IP blueheads in general, which are attacked by various common predatory fishes, including serranids, synodontids, carangids and scombrids [37], (DRR pers obs). Further, juvenile wrasse-blennies do not resemble potentially ”protected” juvenile blueheads. Thus the idea of a Batesian mimicry based on a “protected” status of IP blueheads [3], [31] is at most weakly supported. In the absence of aggressive or Batesian mimicry the association of wrasse-blenny and blueheads might still provide “Social mimicry” benefits: reduced predation risk to the blenny due to its rarity and inconspicuousness in aggregations of IP blueheads.

Could the resemblance of *H. simulus* and IP blueheads be coincidental? That should not be ruled out. First, many of the unusual morphological characteristics of *Hemiemblemaria* are shared to varying degrees with those other members of its clade that show a tendency to free-swim using their pectoral fins. All those characteristics, plus a swim bladder, may have evolved to facilitate use of mid-water habitat rather than evolving specifically to enhance a resemblance of *H. simulus* to IP blueheads. The coloration of *H. simulus* adults shares basic features with the coloration of many nemophine blenniids that are free-swimming like *H. simulus*, and like it have swim bladders: yellow and white bodies, often with one or more dark stripes, sometimes with a single mid-lateral stripe that may be solid or broken into a series of blotches [22], [25], [27]. Coloration of this general type is not unusual among other elongate reef fishes that free-swim in near-bottom habitats, including various labrids, haemulids, mullids, and gobiids (DRR pers. obs). Both the repeated redevelopment of adult swim bladders in different blennioid families and coloration like that of *Hemiemblemaria* may represent morphological elements generally used by blennioids that adopt a free-swimming lifestyle.

In the absence of an evolved mimetic relationship a social-trap could lead to the blenny associating with schools of the coincidentally similar wrasse. If that behavior is rewarded through enhanced access to food shared with the schooling wrasse (free swimming crustaceans) due to reduced predation risk on a rare, inconspicuous blenny in a wrasse school, learning could lead to the blenny regularly associating with the wrasse. Detailed fieldwork revealed previously unsuspected complexity in the mimicry of the cleaner wrasse by *Aspidontus taeniatus* [6]. Clarification of the relationship between the wrasse-blenny and the wrasse will require a similarly intensive investigation of the behavioral ecology of the blenny that assesses different explanations for their association.

#### Conclusion

There is equivocal evidence of mimicry in this case, which is also consistent with the social-trap hypothesis (see Table 1). If there is mimicry it is more likely it is social rather than aggressive or Batesian as originally proposed.

### III Multiple Hamlets, *Hypoplectrus* spp., and Various Perciform Fishes

*Hypoplectrus* is a genus of small, predatory groupers endemic to the tropical Northwest Atlantic. It has 16 named ‘species’ [3], [39]-[42], eight of which have been proposed as aggressive mimics of different reef fishes [3], [42], [43]. Below I present information on the only four species for which there are behavioral observations relating to the mimicry hypothesis. Relevant information on the coloration and behavior of five other species of “mimic” hamlets and their “models” is summarized in Appendix S1, together with general information on the coloration of hamlets.

#### Hypoplectrus indigo

**Figure 3:**
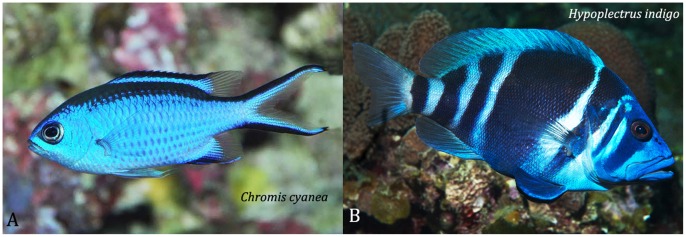
The indigo hamlet, *Hypoplectrus indigo,* and its supposed model, the blue chromis, *Chromis cyanea*. Photos: A - DR Robertson; B - G Stoyle.

The indigo hamlet has a dark blue body and fins plus 7 dark blue-black bars on its head and body (see Figure 3, and [33], [39], [40], [44]). The distribution and intensity of the blue ground coloring of the body and fins varies among individuals, and some fish have thin black stripes along the top and bottom edges of the tail fin. *H. indigo* was not included in the original group of seven “mimic” hamlets proposed by Randall and Randall [3] or Thresher [42]. This species commonly feeds on juveniles of *Chromis cyanea* (mainly) and *C. insolata* [45], [43]. The former are plain iridescent blue and the latter are iridescent purplish-blue with a yellow back. Fischer [45] proposed that *H. indigo’s* coloration blends into background of the water column, facilitating the hamlets approach to *C. cyanea*. Whiteman et al [43] in turn suggested that *H. indigo* may be an aggressive mimic of *C. cyanea*; i.e. a predator mimicking its prey. Neither author explained how a deep-oblong dark blue, darkly barred fish might either blend into the mid-water background (as viewed by a chromis?), or mimic a smaller, plain blue fish with an elongate oval body (see Figure 3).

#### Hypoplectrus nigricans

**Figure 4:**
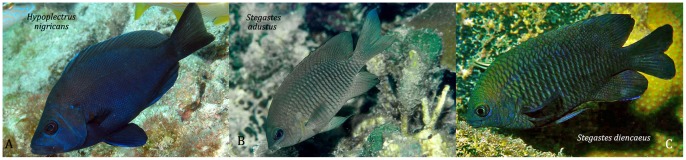
The black hamlet, *Hypoplectrus nigricans,* and its supposed damselfish models, the dusky damselfish, *Stegastes adustus,* and the longfin damselfish, *Stegastes diencaeus.* Photos: A - A Bulanov; B & C - DR Robertson.

“Typical” black hamlets have uniformly dark brown to black bodies and fins. However, some fish have a dark blue cast superimposed on the lower body and fins (see Figure 4), some fish have paler tails with black stripes on the top and bottom edges of the fin, some have a black saddle on the upper caudal peduncle, and the pectorals may be clear or yellow rather than black [33].

Thresher [42] proposed that *H. nigricans* is a mimic of the herbivorous damselfish *Stegastes adustus* (as *S dorsopunicans*), while Randall [5] considered it to mimic both *S. adustus* and the similarly colored *S diencaeus* (see Figure 4). *S. adustus* is pale to mid-brown and *S. diencaeus* blackish brown. The juveniles of both those *Stegastes* species are very differently colored to both conspecific adults and to hamlets in general (e.g. see [33], [44]). Both those damselfishes reach their greatest densities in water <10 m deep [46] where *H. nigricans* is most abundant [42], [45], [47].

Fischer [45] made intensive observations on *H. nigricans* and noted no behavioral interactions between it and the damselfishes or other species indicative of mimicry. He suggested that the coloration resemblance between *H. nigricans* and the two *Stegastes* species is coincidental, and the result of independent selection for background-matching crypsis in each taxon. Aguilar-Perera [48] described geographic variation in color, shape and size of *H. nigricans*, with fish in the northwest Caribbean being uniformly black, with short, blunt fins, while those in Puerto Rico are grey with yellow eyes, and have longer, more pointed fins (see also [33], [40]). If the black pattern is cryptic [45], then the geographic variation described by Aguilar-Perera [48] indicates that the species may be less cryptic at some locations than others.

According to Thresher [42] *H. nigricans* and *S. adustus* show parallel geographic variation in coloration, with the Jamaican population of both having yellow bellies, and pelvic, anal and tail fins. However, at Montego Bay, Jamaica, ∼50 km from Thresher’s study site, *S. adustus* have the same uniform grey-brown color they have elsewhere in the Caribbean area (DRR pers obs, at Montego Bay, Florida, Bermuda, the Bahamas, Panama, Curacao, Venezuela, Barbados, and Puerto Rico). Further, the Jamaican coloration described for the hamlet and damselfish by Thresher [42] fits other hamlets (*H. aberrans* or *H. chlorurus*) and *Stegastes variabilis*. When color differences are the defining characteristic of most hamlet “species” (see Appendix S1), whether to call hamlets with different color patterns intraspecific geographic variants rather than different species becomes a semantic issue.

#### Hypoplectrus puella

**Figure 5:**
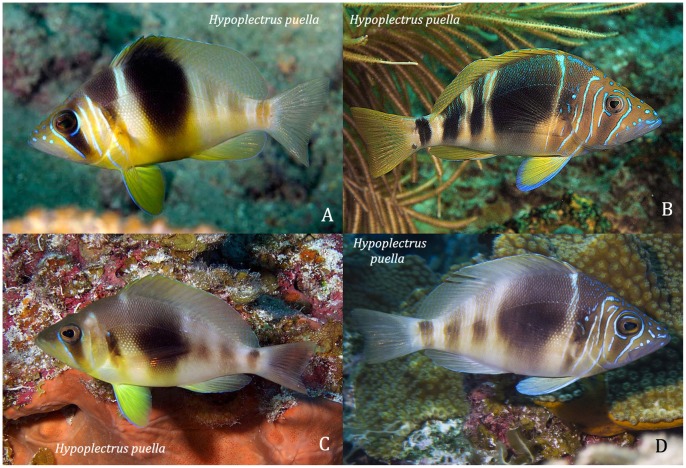
Four color variants of the barred hamlet, *Hypoplectrus puella.* Photos: A - J DeMarino; B - K Bryant; C - C Cox (Mexico Beach Artificial Reef Association); D - J Garin.

The barred hamlet is the commonest and most widespread member of the genus. It has a pale yellowish to tan head and body with up to 7 dark brown bars that vary in their occurrence, intensity and vertical extent (see Figure 5). The predominant colors are browns and yellows, but some individuals have blue tones. The large, conspicuous pelvics fins vary from yellow to dark bluish brown. The head and, occasionally, the body may have fine vertical iridescent blue lines (see Figure 5, and [33], [44], www.fishdb.co.uk and www.reefguide.org ). Thresher [42] described geographic variation in coloration of *H puella*: the proportional abundances of four different barring patterns varied in different parts of the geographic range. If *H. puella’s* barred coloration is cryptic [42], then such geographic variation indicates that it may be less cryptic at some locations than others.

In Panama *H. puella* regularly acts as a follower of demersal feeding schools of a small parrotfish [49], [50]. *H. puella*, and other predatory fishes, take advantage of the fact that the compact parrotfish schools displace and mobilize prey organisms as they move slowly across the substrate pillaging the algal gardens of territorial damselfishes. Barred hamlets following parrotfish schools attack the small disturbed and distracted prey and have a much higher rate of predatory strikes than do solitarily hunting individuals [50]. In both the hamlet and parrotfish brown hues predominate, with patterns composed of stripes and blotches in the elongate parrotfish, which is more dully colored when in schools than when defending territories [49], (DRR pers obs), and bars in the deep-bodied hamlet. Here a non-mimetic hamlet associates with a parrotfish that resembles it only in having generally similar color hues.

#### Hypoplectrus unicolor

**Figure 6:**
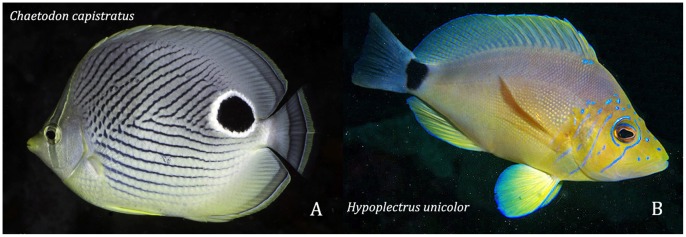
The butter hamlet, *Hypoplectrus unicolor,* and its supposed model, the foureye butterflyfish, *Chaetodon capistratus*. Photos: A & B - J Garin.

Thresher [42] proposed that butter hamlet *H. unicolor* is mimic of the foureye butterflyfish *Chaetodon capistratus* (see Figure 6), based on similarities in their color patterns and general shape, common usage of shallow habitats, the relative rarity of *H. unicolor* and differences in their diets: mobile benthic animals in the hamlet versus sessile benthic invertebrates in the butterflyfish. Butter hamlets that most resemble *C. capistratus* have a whitish body, yellowish fins, and a triangular black saddle on the upper half of the caudal peduncle. However, the body of this hamlet varies from whitish-tan to yellow, and may have up to 4 additional markings that reduce its resemblance to the chaetodon: (i) dark blotches under or before the main tail-base saddle, which may be replaced by a black bar across the entire tail base, (ii) a large, black blotch ringed with blue on one or both sides of the snout, (iii) many blue vertical lines on the head, and, less frequently the body, and (iv) faint dark bars on the body in similar to those seen in *H. puella* [33], [40], [42], [44], [51], [52], www.fishdb.co.uk, www.reefguide.org ). The occurrence of the large lateral snout blotch also varies geographically in this species [42], [52].

*Chaetodon capistratus* has an oval body, versus oblong in the hamlet. The general form of *C. capistratus’* coloration resembles that of *H. unicolor*. However, the rear black blotch is a ringed ocellus located further forwards on the body in *C. capistratus*, and each species lacks prominent color elements found in the other, eg. a chevron pattern of thin black lines on the body of the butterflyfish. While there is local and geographic variation in the color pattern of *H. unicolor* [42], [52], there is no indication of equivalent variation in *C. capistratus*, which look essentially the same throughout its range (e.g. www.fishdb.co.uk, www.reefguide.org, [33]) The color pattern of small juvenile *C. capistratus* differs from that of conspecific adults, and from that of adults and juveniles of *H, unicolor* and other hamlets (e.g. [33], [44]).

Puebla et al [8] provided the first description of a behavioral relationship between the butter hamlet and *C. capistratus*. They found that the hamlet had a predatory-strike rate much higher when closely following feeding pairs of *C. capistratus* and attacking small organisms disturbed by the latter than when feeding alone, although the hamlet spent only 10% of its time associating with the butterflyfish. In comparison, *H. puella* in the same area rarely associated with *C. capistratus*, and showed a non-significantly elevated predatory-strike rate when doing so.

#### The hamlet data in relation to the nine predictions

Observations of behavioral interactions relating to mimicry have been made on only two of the seven hamlets originally described as mimics by Randall and Randall [3] and Thresher [42], *H. nigricans* and *H. unicolor*. In a detailed study of the behavioral ecology of *H. nigricans,* Fischer [45] found no support for mimicry by this species, and suggested there was a coincidental similarity related to background matching coloration. *H. unicolor* and *H. indigo* are the only hamlets actually known to behave in a manner consistent with a mimetic relationship. How do information on hamlets in general relate to the nine predictions of the mimicry hypothesis?

#### Quality of the resemblance

To this human observer the resemblance of each of the eight “mimic” hamlets to its “model” is best in *H. gemma*, good in *H. unicolor*, and varies from approximate to marginal in the remainder. *H. unicolor* does resemble *C. capistratus* in general features of shape, size, and basic coloration, but not in details of the color pattern. Resemblances are best when a “model” and a nearby “mimic” are viewed at a distance, whereas near-distance similarity likely is more important if it affects hamlet predation potential. As far as is known small juveniles of the different hamlet species all have a similar coloration (see Appendix S1), which is different to the adult coloration and also unlike the coloration of equivalent sized juveniles of any of the putative “models” for the different hamlet species. Thus any potential for mimicry likely would be restricted to subadult and adult hamlets. All “mimetic” hamlets, except apparently *H. gemma*, display local variation in their coloration, and hence in the degree of resemblance to their “models”.

#### Parallel geographic variation in the resemblance

Distinct geographic variation in the coloration is present in *H. unicolor, H. nigricans*, and *H. indigo*, but not in the “model” of each. *H. puella* and *H. nigricans* display geographic variation in coloration that suggests that, if their coloration is cryptic, they are more cryptic at some locations than others. Thus the limited information on geographic variation in hamlet coloration indicates it occurs in both “mimic” and non-mimic hamlets and is not related to mimicry, nor, perhaps, to crypsis.

#### Resemblance characteristics unusual for the taxon

None of the “mimic” hamlets display unusual coloration features that are not found in non-mimic hamlets, and all elements of the coloration of each “mimic” hamlet are seen in multiple non-mimetic hamlet species (see Appendix S1). *H. unicolor* and other “mimic” hamlets display much the same degree of color pattern variation as other hamlets. *H. unicolor* has a typical hamlet shape, although it may be marginally deeper bodied than some other species. Randall & Randall [3] suggested that *H. gemma* has a slightly more elongate body than other hamlets, which would enhance its resemblance to its elongate oval “model” *Chromis cyanea* (see Appendix S1). However, small hamlets are more elongate than large individuals of the same species, and some adults of *H. gemma* are less elongate than others. Body shapes of different “mimetic” and other hamlets need a quantitative reassessment that takes into account allometric growth. No taxonomically unusual behavior has been recorded for any “mimetic” hamlet that might enhance its resemblance to its model.

#### Target perceives model and mimic as similar, and identifies model’s status

Hamlets eat mysids, decapod shrimp, crabs, squillid stomatopods and fishes [37]. Thresher [42] proposed that the primary targets for hamlet mimicry are crustaceans, the most important component of the diets of most species, and that imprecise mimetic resemblances of hamlets to their “models” reflected relatively poor visual capabilities of those targets; ie imprecise similarity is sufficient. What does a mysid/shrimp/crab/squillid stomatopod see when it sees a hamlet, or its “model”, or another small fish? Squillids lack the highly developed vision of other stomatopods [53], [54]. Members of those four groups seem to be aware mostly of movement, and, due to relatively poor resolving power and a simple color sense at best, likely respond visually to different species of fishes in much the same way (NJ Marshall, pers com, July 2012). That indicates those crustaceans perceive not only hamlets and their “models” but also a broad range of other fishes as similar, and are visually incompetent to discriminate between them and to determine the benign status of hamlet “models”. The avoidance reactions of small crustaceans to feeding activities of the parrotfish and butterflyfish that facilitates attacks on those prey by *H. puella* and *H. unicolor* is consistent with those crustaceans being unable to determine the harmless nature of the former.

Two “mimetic” hamlets, *H. nigricans* and *H. indigo,* feed heavily on fish: ∼30–40% and 90% of their identifiable stomach contents respectively [37], [43], [45]. In addition, fishes also make up about 10–25% of the identifiable stomach contents of *H. chlorurus*, *H. puella* and *H. unicolor* [37], [43]. The resemblance of the “mimetic” hamlet that specializes in preying on fish, *H indigo*, to its potential model, the fish it eats, is sufficiently vague that it was not among the original group of hamlets labeled as mimics due to their similarity to other fishes. Caribbean reef fishes, including potential targets of some “mimic” hamlets, can readily distinguish those hamlets from their models [42], and recognize the different ecological and threat status of each.

#### Relative abundance

The prediction that the mimic should be much less common than its model is met in most “mimic” hamlets, including *H. unicolor* The exceptions are *H. guttavarius* and *H. aberrans* with one of each species’ “models” (see Appendix S1).

#### Association in space

All “mimic” hamlets overlap in space at both large and small scales with their models. However, close behavioral associations between model and “mimic” are known only for *H. unicolor* and *H. indigo*. Predatory reef fishes of a variety of taxa generally respond to the activity of large organisms (fishes and divers) on the substratum by approaching and attacking prey distracted and mobilized by the disturbance. They often act as followers of dissimilar schooling species and other species that disturb the substratum [49], [50], [55], [56], [57], [58], (DRR pers obs,). The non-mimetic barred hamlet regularly follows feeding schools of parrotfishes [50] and occasionally follows feeding *C. capistratus* [8]. Thus *H. unicolor’s* feeding association with *C. capistratus* represents an example of a phenomenon that is widespread among predatory reef fishes, and that occurs in both “mimic” and other hamlets. The distinguishing feature of these feeding associations of *H. unicolor* and *H. puella* with other fishes is that in each case the hamlet follows a species that has similar color hues. Similarly, *H. indigo’s* association with two *Chromis* species is also linked to their shared blue color.

#### Diets of “models” and “mimics”

All putative models of predatory “mimic” hamlets are harmless to hamlet prey.

#### Evidence of success of mimicry

In only one case is there any evidence of a reward to a hamlet that is could be attributed to a target confounding the hamlet with its “model”. In that *H. indigo* selectively preys on young (ie. naïve) individuals of its own putative model [45]. However, the resemblance of *H. indigo* to its supposed model is vague and fish can readily distinguish hamlets from their models [42].

#### Evidence of benefits due to resemblance

The increased strike rate of *H. unicolor* following the *C. capistratus*, a likely indicator of predation rate, is consistent with a resemblance benefit. However, that increased predation rate could also simply be due to increased access to prey distracted and mobilized by the disturbance of the feeding butterflyfish regardless of any resemblance. *H. puella* and other predatory fishes have similarly elevated predatory strike rates while following schools of parrotfishes to which they have little or no resemblance. Consistent predation on *C. cyanea* by *H. indigo*, but apparently not other hamlets, does represent evidence of benefits due to a resemblance, except that the resemblance is poor and prey fish most likely can identify the predatory status of hamlets [42].

#### Mimicry versus social-traps among the hamlets

To the human observer the resemblances of the various “mimetic” hamlets to each of their “models” is not very precise, much less so in some cases than others. Various hypotheses have been proposed to account for imprecise mimicry. In addition to the work of Caley and Schluter [59] on Batesian mimicry in reef fishes, research on the origin and maintenance of imprecise mimicry has focused on Batesian mimicry among insects [20], [60]-[62], and snakes [63], [64], as well as floral mimicry in orchids (e.g. [65]), and has been largely predicated on the assumption that the imprecise resemblances under consideration are indeed mimetic. Among the various hypotheses proposed to account for imprecise mimicry ([20] for summary), those that seem potentially relevant to the imprecise resemblances of the “mimic” hamlets include (1) the illusion hypothesis (human perceptions differ from those of the mimicry target); (2) the strength of selection hypothesis (selection is weaker on imperfect mimics); (3) the multi-model hypothesis (the mimic is intermediate in appearance between multiple models); (4) the constraints hypothesis (tradeoffs or phylogenetic constraints limit the capacity for developing precise resemblance); and (5) the disequilibrium hypothesis (the mimicry has broken down).

Thresher [42] proposed that the imprecision in the resemblance of mimetic hamlets to their models is illusory, because the mimicry targets, small crustaceans, have more limited visual capabilities than human observers. However, crustacean “targets” likely are visually incompetent to distinguish not only between model and “mimic”, but also between them and other fishes, and treat them all as a threat. Fishes, which have much better eyesight than small crustaceans, can readily distinguish between hamlets and their “models” and are aware of the threat status of hamlets [42]. Fishes represent a significant prey type for most hamlets and are a major type for at least two species, which do not resemble their “models” more precisely than other hamlets that prey primarily on crustaceans. Thus there is little support for the illusion hypothesis in hamlets. Among the remaining four hypotheses outlined above the strength-of-selection and constraints hypotheses may have the most relevance to the hamlet situation. For example, constraints may limit the extent to which the shape of predatory hamlets may be modifiable to approximate that of differently shaped models that vary from midwater planktivores to benthic herbivores. There is insufficient information across the full range of hamlet species to assess the remaining hypotheses (strength of selection, disequilibrium, multi-model), although none seems a good candidate. Thus, while some aspects of the interspecific associations involving *H. indigo* and, particularly, *H. unicolor* are consistent with mimicry, the supporting evidence is suggestive rather than decisive, and other evidence (e.g. the visual incompetence of crustacean prey) is counter-indicative (see Table 1).

The behavioral associations between each of *H. unicolor* and *H. indigo* and its model, the only ones known to exist among eight cases of supposed hamlet mimicry, are consistent with the social-trap hypothesis, as follows: Hamlets are day-active, visually oriented organisms that strongly select like-colored mates from among a local pool of many differently colored hamlet “species” [8], [52], [66]–[69]. Due to this strong, color-based social response, hamlets may be “socially” attracted to other similarly colored species of fish. With the butter hamlet a coincidental resemblance of *Chaetodon capistratus* to the hamlet could induce the hamlet to approach the feeding chaetodon. As with parrotfish schools followed by *H. puella*, and other species followed by other predatory fishes, feeding *C. capistratus* disturb small mobile benthic crustaceans and fishes at a relatively high rate as they actively move about inspecting and taking a few bites here and a few there on the substratum. This would greatly facilitate predation by *H. unicolor*, and could induce them to follow the butterflyfish. The learned rewards from that following behavior could reinforce its further development in the hamlet. *C. capistratus* is a common, conspicuous species that often feeds in pairs or groups and is likely to repeatedly attract the attention of butter hamlets, facilitating the development of a regular association between the two. Thus two pre-existing behaviors common to predatory fishes, including non-mimic hamlets, could predispose *H. unicolor* to develop an association with *C. capistratus*: (i) a tendency to follow unrelated fishes whose activities provide enhanced access to prey, and (ii) a strong social attraction to like colored fishes. The regular associations of brown *H. puella* with schools of a brown parrotfish, (but rarely with yellow *C. capistratus*), and of blue *H. indigo* with blue *Chromis* spp, also are consistent with the action of a social-trap based on coincidental color similarity.

The broad matrix of variation in coloration among and within hamlet species (see Appendix S1) provides for a large number of possible combinations of pattern and color elements, as an internet search for images of “hamlets” readily shows. Hence, it not surprising that some hamlet species coincidentally resemble one or more of the many small, similar sized fishes of different taxa with which hamlets share Caribbean reefs (cf [52]). Nor is it surprising that other hamlets fail to do so. In fact the model of each of the original seven “mimic” hamlets was identified not through behavioral interactions between the two suggestive of mimicry, but on the basis of its general appearance, habitat use, abundance and diet, i.e. because it satisfied those four predictions of the mimicry hypothesis. The social-trap hypothesis is consistent with all aspects of hamlet “mimicry”, including those aspects that are not readily consistent with mimicry. For the hamlets the social-trap hypothesis offers a more parsimonious explanation than the mimicry hypothesis, and makes fewer assumptions about resemblances and the sensory capabilities of different potential “targets”.

#### Conclusions

In five of eight cases there is no direct evidence of a mimetic relationship between a hamlet and another fish species, as relevant behavioral observations are completely lacking. In one other case (*H. nigricans*) behavioral observations provided no support for a mimetic relationship. Although In the remaining two cases (*H. unicolor* and *H. indigo*) observed behaviors of hamlets are consistent with mimicry, various predictions of the mimicry hypothesis are not met and seem unlikely to be met. On the other hand all observations are consistent with the social-trap hypothesis, which can better explain all the information currently available on hamlet relationships to other, trophically different reef fishes that are broadly similar to them in color and form (see Table 1).

### General Conclusions

This analysis indicates that there is strong support for aggressive mimicry in only one of three well known cases of this phenomenon among tropical reef fishes. In one other case the evidence is inconclusive and also consistent with a coincidental resemblance of “model” and “mimic”. In the third case, involving eight species of *Hypoplectrus*, none of the more important predictions of the mimicry hypothesis are fulfilled. There the existing evidence is more consistent with a coincidental resemblance of “model” and “mimic” leading to a social attraction of “mimic” to a fish that resembles itself, and the development of a behavioral association through behaviors typical of many predatory reef fishes. My examination of color photographs of ∼50 “mimic-model” pairs listed by Moland [2] indicates that resemblances of shape and coloration between each ‘mimic’ and ‘model’ are sufficiently imprecise in about half those cases that those resemblances could be coincidental. Only a handful of cases of supposed aggressive mimicry other than the three discussed here have been examined carefully in any depth, particularly with experimental manipulations in the field [7], [9], [12], [14], [70]–[73]. Little work beyond descriptions of interspecific similarities has been done on other major types of mimicry ascribed to reef fishes – Batesian, Mullerian and Social mimicry [2], [5]. Caley & Schluter’s [59] study of imprecise Batesian mimicry is a notable exception. Some imprecise similarities described for Batesian, Mullerian and Social mimicry could reflect coincidental similarity, and, in some cases, the action of social traps. Social traps may be particularly important in cases of supposed Social mimicry, as schooling among diurnal reef fishes relies on strong, visually mediated social attraction between conspecifics. For example, many unrelated species of fishes in different parts of the world that school in midwater or on sand bottoms are elongate and silvery, an often coincidental similarity that could predispose some of them to school together.

It is clear from the results of the present analyses and a dearth of comprehensive information that most associations of look-alike species need skeptical re-evaluation that examines which of various alternative explanations best accounts for new data relating to a series of pointed questions about the nature of the relationship. Coincidental resemblance of associating unrelated species represents the default condition that needs to be effectively discounted to establish the case for a mimetic relationship, something that may be quite difficult to do in many cases of imprecise resemblance. Social traps based on coincidental, generalized resemblance may often represent an end point that supports a behavioral association between two species. However, such traps could well set the stage for the evolution of mimicry, by initially promoting the development of an interspecific association that then becomes honed by selection through the agency of a newly involved mimicry target.

## Supporting Information

Figure S1 **The blue hamlet** ***Hypoplectrus gemma*** **and its supposed model, the blue chromis** ***Chromis cyanea***. Photos: A & B - DR Robertson.
(TIF)

Figure S2 **The yellowbelly hamlet** ***Hypoplectrus aberrans*** **and its supposed model, the cocoa damselfish** ***Stegastes variabilis*** **.** Photos: A - F Charpin; B - DR Robertson.
(TIF)

Figure S3 **The yellowtail hamlet** ***Hypoplectrus chlorurus*** **and its supposed model, the yellowtail damselfish** ***Microspathodon chrysurus.*** Photos: A - C Shipley; B - DR Robertson.
(TIF)

Figure S4 **The shy hamlet** ***Hypoplectrus guttavarius*** **and its supposed model, the rock beauty angelfish** ***Holacanthus tricolor*** **.** Photos: A - F Charpin; B – J Lyle.
(TIF)

Figure S5 **The tan hamlet** ***Hypoplectrus randallorum*** **and its supposed model, the threespot damselfish** ***Stegastes planifrons.*** Photos: A - P Lobel; B - DR Robertson.
(TIF)

Appendix S1 **General features of hamlet coloration, and five additional supposed mimetic hamlets.**
(DOC)

## References

[1] Wickler W (1968) Mimicry in plants and animals. New York: McGraw Hill, 475 p.

[2] Moland E, Eagle JV, Jones GP (2005) Ecology and evolution of mimicry in coral reef fishes. Oceanogr Mar Biol Ann Rev 43: 455–482.

[3] Randall JE, Randall HA (1960) Examples of mimicry and protective resemblance in tropical marine fishes. Bull Mar Sci 10: 444–480.

[4] Randall JE, McCosker JE (1993) Social mimicry in fishes. Rev Franc Aquariol 20: 5–8.

[5] Randall JE (2005) A Review of Mimicry in Marine Fishes. Zool Stud 44: 299–328.

[6] Kuwamura T (1983) Reexamination on the aggressive mimicry of the cleaner wrasse *Labroides dimidiatus* by the blenny *Aspidontus taeniatus* (Pisces: Perciformes). J Ethol 1: 22–33.

[7] Eagle JV, Jones GP (2004) Mimicry in coral reef fishes: ecological and behavioural responses of a mimic to its model. J Zool 264: 33–43.

[8] Puebla O, Bermingham E, Guichard F, Whiteman E (2007) Colour pattern as a single trait driving speciation in *Hypoplectrus* coral reef fishes? Proc R Soc B 274: 1265–1271. 10.1098/rspb.2006.0435 PMC2176177 17360287

[9] Cheney K L, Côté I M (2005) Frequency-dependent success of aggressive mimics in a cleaning symbiosis. Proc R Soc B 272, 2635–2639. 10.1098/rspb.2005.3256 PMC1559983 16321786

[10] Cheney K L, Grutter AS, Marshall NJ (2008) Facultative mimicry: cues for colour change and colour accuracy in a coral reef fish. Proc R Soc B 275: 117–122. 10.1098/rspb.2007.0966 PMC2596177 17986437

[11] Cheney KL, Marshall N J (2009) Mimicry in coral reef fish: how accurate is this deception in terms of colour and luminance? Behav Ecol 20: 459–468.

[12] Rainey MM (2009) Evidence of a geographically variable competitive mimicry relationship in coral reef fishes. J Zool 279: 78–85.

[13] Cheney KL (2012) Cleaner wrasse mimics inflict higher costs on their models when they are more aggressive towards signal receivers. Biol Lett 8: 10–12. 21865244 10.1098/rsbl.2011.0687 PMC3259977

[14] Munday PL, Eyre PJ, Jones GP (2003) Ecological mechanisms for coexistence of colour polymorphism in a coral-reef fish: an experimental evaluation. Oecologia 137: 519–526. 13680346 10.1007/s00442-003-1356-7

[15] West-Eberhard MJ (1984) Sexual selection, competitive communication and species-specific signals in insects. In: Lewis T, editor. Insect Communication. New York: Academic Press. 283–324.

[16] Christy J (1995) Mimicry, mate choice, and the sensory trap hypothesis. Amer Nature 146: 171–181.

[17] Edwards DP, Wu DW (2007) The roles of sensory traps in the origin, maintenance, and breakdown of mutualism. Behav Ecol Sociobiol 61: 1321–1327.

[18] Sazima I (2002) Juvenile grunt (Haemulidae) mimicking a venomous leatherjacket (Carangidae), with a summary of Batesian mimicry in marine fishes. Aqua 6: 61–68.

[19] Sazima I (2002) Juvenile snooks (Centropomidae) as mimics of mojarras (Gerreidae), with a review of aggressive mimicry in fishes. Envir Biol Fish 65: 37–45.

[20] Penney HD, Hassall C, Skevington JH, Abbott KR, Sherratt TN (2012) A comparative analysis of the evolution of imperfect mimicry. Nature 483: 461–464. 22437614 10.1038/nature10961

[21] Sikkel PC, Hardison PD (1992) Interspecific feeding association between the goatfish *Mulloides martinicus* (Mullidae) and a possible aggressive mimic, the snapper *Ocyurus chrysurus* (Lutjanidae). Copeia 1992: 914–917.

[22] Springer VG, Smith-Vaniz WF (1972) Mimetic Relationships Involving Fishes of the Family Blenniidae. Smith Contrib Zool 112: 1–36.

[23] Hastings PA, Springer VG 2009 Recognizing diversity in blennioid fish nomenclature (Teleostei: Blennioidei). Zootaxa 2120: 3–14.

[24] Cheney KL, Eckes MJ (2008) Cleaner fish clean cleaner mimics. Coral Reefs 27: 527.

[25] Smith-Vaniz WF (1987) The saber-toothed blennies, tribe Nemophini (Pisces: Blenniidae): an update. Proc Acad Nat Sci Philad 139: 1–52.

[26] Russell BC, Allen GR, Lubbock HR (1976) New cases of mimicry in marine fishes. J Zool 180: 407–423.

[27] Smith-Vaniz WF (1976) The saber-toothed blennies, tribe Nemophini (Pisces: Blenniidae). Monogr Acad Nat Sci Philad 19: 1–196.

[28] Grutter AS, Bshary R (2003) Cleaner wrasse prefer client mucus: support for partner control mechanisms in cleaning interactions. Proc.R Soc B (Suppl.) 270: S242–S244. 10.1098/rsbl.2003.0077 PMC1809949 14667394

[29] Shand J (1997) Ontogenetic changes in retinal structure and visual acuity: a comparative study of coral-reef teleosts with different post-settlement lifestyles. Envir Biol Fish 49: 307–322.

[30] Marshall NJ (2000) The visual ecology of reef fish colours. In: Espmark Y, Amundsen T, Rosenqvist G, editors. Animal Signals: Signalling and Signal Design in Animal Communication: Trondheim, Tapir Academic Press. 83–120.

[31] Longley WH, Hildebrand SF (1940) New genera and species of fishes from Tortugas, Florida. Pap Tortugas Lab 32: 223–285.

[32] Feddern HA (1965) The spawning, growth, and general behavior of the bluehead wrasse, *Thalassoma bifasciatum* (Pisces: Labridae). Bull Mar Sci 15: 896–941.

[33] Reefnet Inc (2007) Reef Fish ID: Florida, Caribbean, Bahamas. DVD version 4.0. www.reefnet.ca.

[34] Stephens JS (1963) A revised classification of the blennioid fishes of the American family Chaenopsidae. Univ. Calif Publ. Zool. 68: 1–165.

[35] Lin H-C, Hastings PA (2011) Evolution of a Neotropical marine fish lineage (Subfamily Chaenopsinae, Suborder Blennioidei) based on phylogenetic analysis of combined molecular and morphological data. Mol Phyl Evol 60: 236–248. 10.1016/j.ympev.2011.04.018 21550409

[36] Kells V, Carpenter K (2011) A field guide to coastal fishes from Maine to Texas. Baltimore: Johns Hopkins University Press.

[37] Randall JE (1967) Food habits of reef fishes of the West Indies. Stud Trop Oceanogr 5: 665–847.

[38] DeLoach N (1999) Reef Fish Behavior: Florida, Caribbean, Bahamas. Jacksonville: New World Publishers, 359 p.

[39] Randall JE (1998) Caribbean reef fishes. 3^rd^ Ed. Neptune City: TFH Publications, 368 p.

[40] Lobel PS (2011) A review of the Caribbean hamlets (Serranidae, *Hypoplectrus*) with description of two new species. Zootaxa 3096: 1–17.

[41] Victor BC (2012) *Hypoplectrus floridae* n.sp. and *Hypoplectrus ecosur* n. sp., two new Barred Hamlets from the Gulf of Mexico (Pisces:Serranidae): more than 3% different in COI mtDNA sequence from the Caribbean *Hypoplectrus* species flock. J Ocean Sci Found 5: 1–19.

[42] Thresher RE (1978) Polymorphism, mimicry, and the evolution of the hamlets (*Hypoplectrus*, Serranidae). Bull Mar Sci 28: 345–353.

[43] Whiteman EA, Côté I M, Reynolds J D (2007) Ecological differences between hamlet (*Hypoplectrus*: Serranidae) colour morphs: between-morph variation in diet. J Fish Biol 71: 235–244.

[44] Humann P (2008). Reef Fish Identification, Florida, Caribbean, Bahamas 3rd ed. Jacksonville: New World Publications. 481 p.

[45] Fischer EA (1980) Speciation in the hamlets (*Hypoplectrus:* Serranidae): a continuing enigma. Copeia 1980: 649–659.

[46] Waldner RE, Robertson DR (1980) Patterns of habitat partitioning by eight species of territorial Caribbean damselfishes. Bull Mar Sci 30: 171–186.

[47] Aguilar-Perera A (2003) Abundance and distribution of hamlets (Teleostei: *Hypoplectrus*) in coral reefs off southwestern Puerto Rico: support for the multiple- species hypothesis. Carib J Sci 39: 147–151.

[48] Aguilar-Perera A (2004) Variations in Morphology and Coloration in the Black Hamlet, *Hypoplectrus nigricans* (Teleostei: Serranidae). Carib J Sc 40: 150–154.

[49] Ogden JC, Buckman NS (1973) Movements, foraging groups, and diurnal migrations of the striped parrotfish, *Scarus croicensis* Bloch (Scaridae). Ecology 54: 591–596.

[50] Robertson DR, Sweatman HPA, Fletcher EA, Cleland MG (1976) Schooling as a mechanism for circumventing the territoriality of competitors. Ecology 57: 1208–1220.

[51] Barlow GW (1975) On the sociobiology of some hermaphroditic serranid fishes, the hamlets, in Puerto Rico. Mar Biol 33: 295–300.

[52] Domeier ML (1994) Speciation in the serranid fish *Hypoplectrus* . Bull Mar Sci 54: 103–141.

[53] Marshall NJ, KentJ, Cronin TW (1999) Visual adaptations in crustaceans: spectral sensitivity in. In: Archer S, Djamgoz M, Lowe E, Partridge JC, Vallerga S, editors. Adaptive Mechanisms in the Ecology of Vision. London: Kluwer. 285–327.

[54] Marshall NJ, Cronin TW, Kleinlogel S (2007) Stomatopod eye structure and function: a review. Arthro Struct Dev 36: 420–448. 10.1016/j.asd.2007.01.006 18089120

[55] Fishelson L (1977) Sociobiology of feeding behavior of coral fish along the coral reef of the Gulf of Eilat ( = Gulf of Aqaba), Red Sea. Israel J Zool 26: 114–134.

[56] Ormond RFG (1980) Aggressive mimicry and other interspecific feeding associations among Red Sea coral reef predators. J Zool 191: 247–262.

[57] Lukoschek V, McCormick MI (2000) A review of multi-species foraging associations in fishes and their ecological significance. Proc 9th Int Coral Reef Symp, Bali, Indonesia 23–27 October 2000. 1: 467–474.

[58] Sazima C, Grossman A (2005) A non-digging zoobenthivorous fish attracts two opportunistic predatory fish associates. Neotrop Ichthyol 3: 445–448.

[59] Caley MJ, Schluter D (2003) Predators favour mimicry in a tropical reef fish. Proc R Soc B 270: 667–672. 10.1098/rspb.2002.2263 PMC1691296 12713739

[60] Edmunds M (2000) Why are there good and poor mimics? Biol J Linn Soc 70: 459–466.

[61] Sherratt TN (2002) The evolution of imperfect mimicry. Behav Ecol 13: 821–826.

[62] Johnstone RA (2006) The evolution of inaccurate mimics. Nature 418: 524–526. 10.1038/nature00845 12152077

[63] Kikuchi DW, Pfennig DW (2010) Predator cognition permits imperfect coral snake mimicry. Amer Natur 176: 830–834. 20950143 10.1086/657041

[64] Pfennig DW, Kikuchi DW (2012) Competition and the evolution of imperfect mimicry. Current Zool 54: 608–619.

[65] Vereecken NJ, Schiestl FP (2008) The evolution of imperfect floral mimicry. PNAS 105: 7484–7488. 18508972 10.1073/pnas.0800194105 PMC2396721

[66] Fischer EA (1980) The relationship between mating system and simultaneous hermaphroditism in the coral reef fish, *Hypoplectrus nigricans* (Serranidae). Anim Behav 28: 620–633.

[67] Barretto FS, McCartney MA (2007) Extraordinary AFLP fingerprint similarity despite strong assortative mating between reef fish colour morphospecies. Evolution 62: 226–233. 18053072 10.1111/j.1558-5646.2007.00285.x

[68] Whiteman EA, Gage MJG (2007) No barriers to fertilization between sympatric colour morphs in the marine species flock *Hypoplectrus* (Serranidae). J Zool 272: 305–310.

[69] Holt BG, Emerson BC, Newton J, Gage MJG, Coté IM (2008) Stable isotope analysis of the *Hypoplectrus* species complex reveals no evidence for dietary niche divergence. Mar Ecol Prog Ser 357: 283–289.

[70] Côté IM, Cheney KL (2007) A protective function for aggressive mimicry. Proc R Soc B 274: 2445–2448. 10.1098/rspb.2007.0797 PMC2274982 17652064

[71] Cheney KL, Skogh C, Hart NS, Marshall NJ (2009) Mimicry, colour forms and spectral sensitivity of the bluestriped fangblenny, *Plagiotremus rhinorhynchos* Proc R Soc. B 276: 1565–1573. 10.1098/rspb.2008.1819 PMC2660993 19324827

[72] Bshary A, Bshary R (2010) Interactions between sabre-tooth blennies and their reef fish victims: effects of enforced repeated game structure and local abundance on victim aggression. Ethology 116: 681–690.

[73] Cheney KL (2010) Multiple selection pressures apply to a coral reef fish mimic: a case of Batesian-aggressive mimicry. Proc R Soc B 277: 1849–1855. 10.1098/rspb.2009.2218 PMC2871877 20181564

