# Supplementary figures and images for: Who Resembles Whom? Mimetic and Coincidental Look-Alikes among Tropical Reef Fishes
Source: PLoS One. 2013 Jan 25;8(1):e54939. doi: 10.1371/journal.pone.0054939 (PMC3556028; doi:10.1371/journal.pone.0054939)

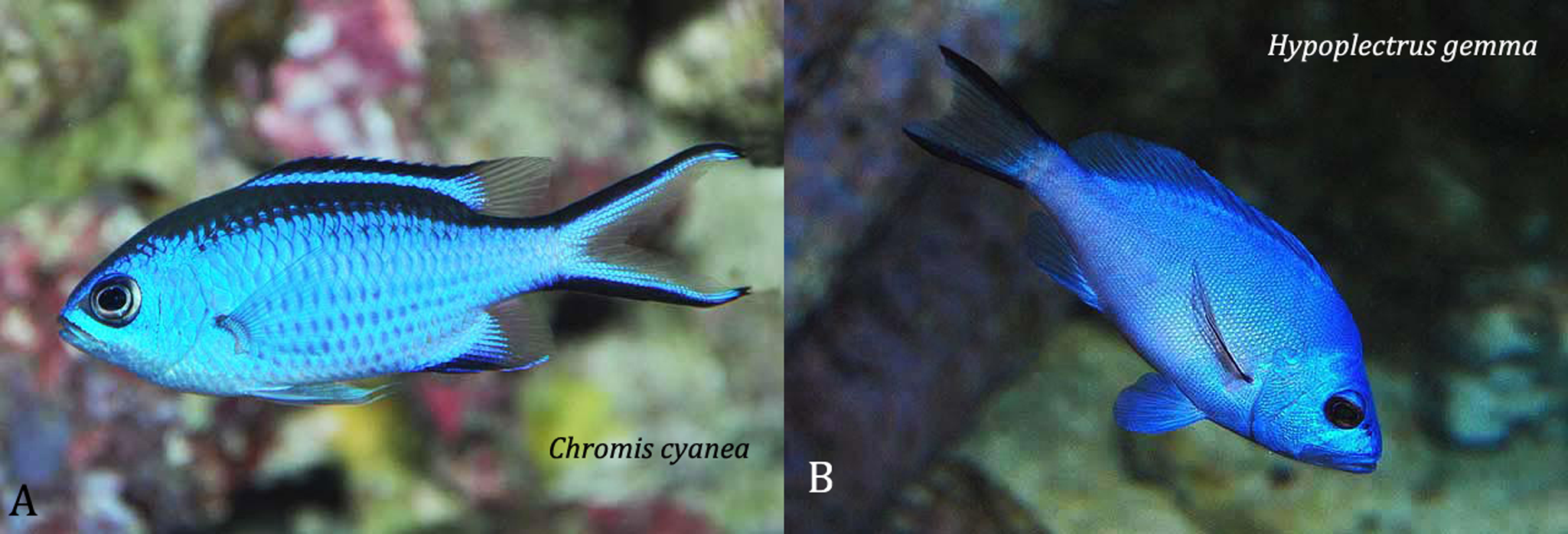

Supplement: Figure S1 — The blue hamlet Hypoplectrus gemma and its supposed model, the blue chromis Chromis cyanea. Photos: A & B - DR Robertson. (TIF) [file pone.0054939.s001.tif]

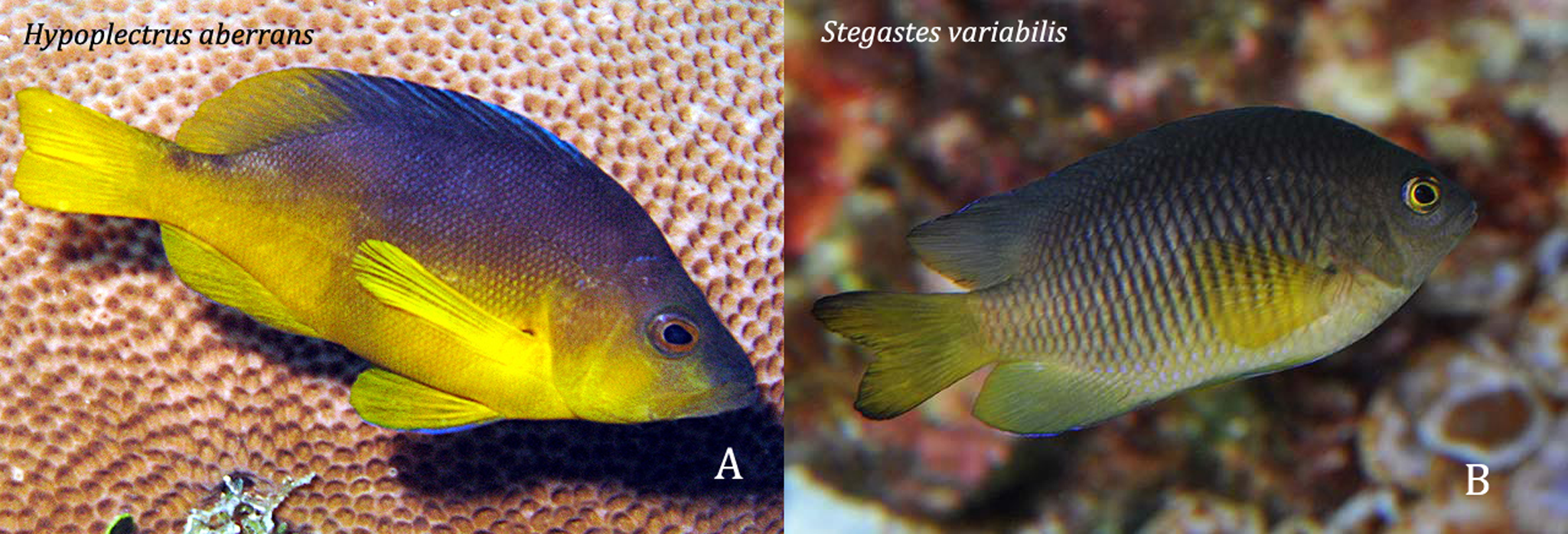

Supplement: Figure S2 — The yellowbelly hamlet Hypoplectrus aberrans and its supposed model, the cocoa damselfish Stegastes variabilis . Photos: A - F Charpin; B - DR Robertson. (TIF) [file pone.0054939.s002.tif]

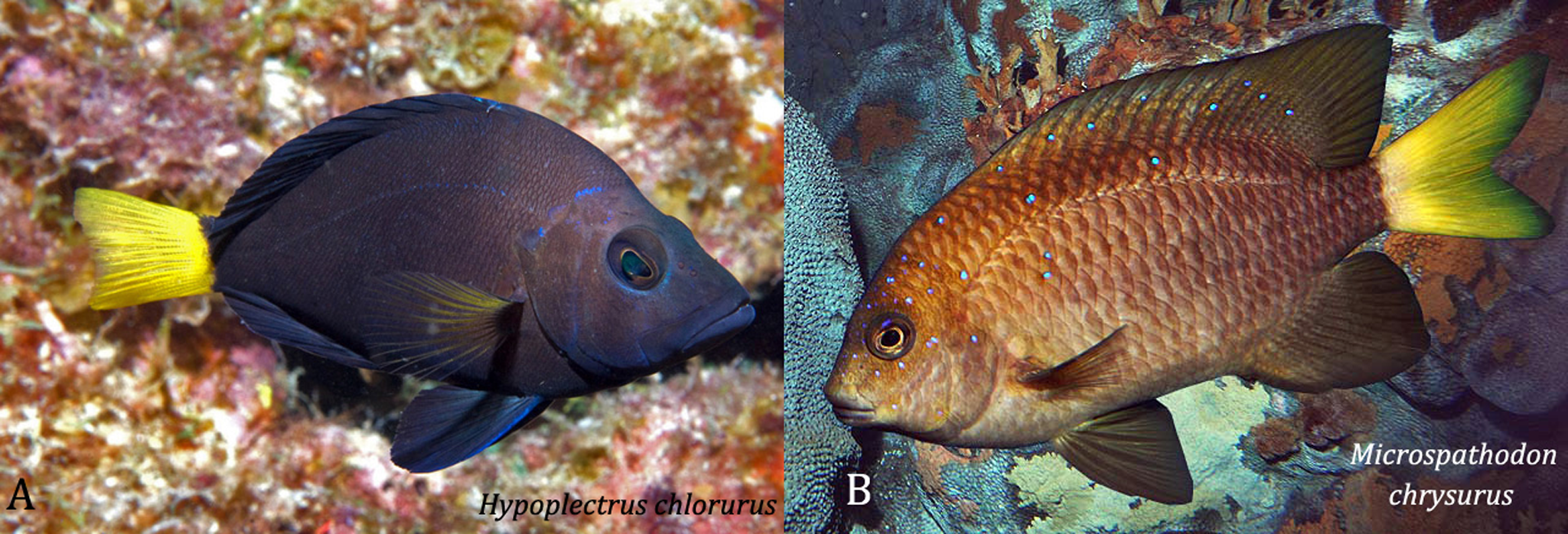

Supplement: Figure S3 — The yellowtail hamlet Hypoplectrus chlorurus and its supposed model, the yellowtail damselfish Microspathodon chrysurus. Photos: A - C Shipley; B - DR Robertson. (TIF) [file pone.0054939.s003.tif]

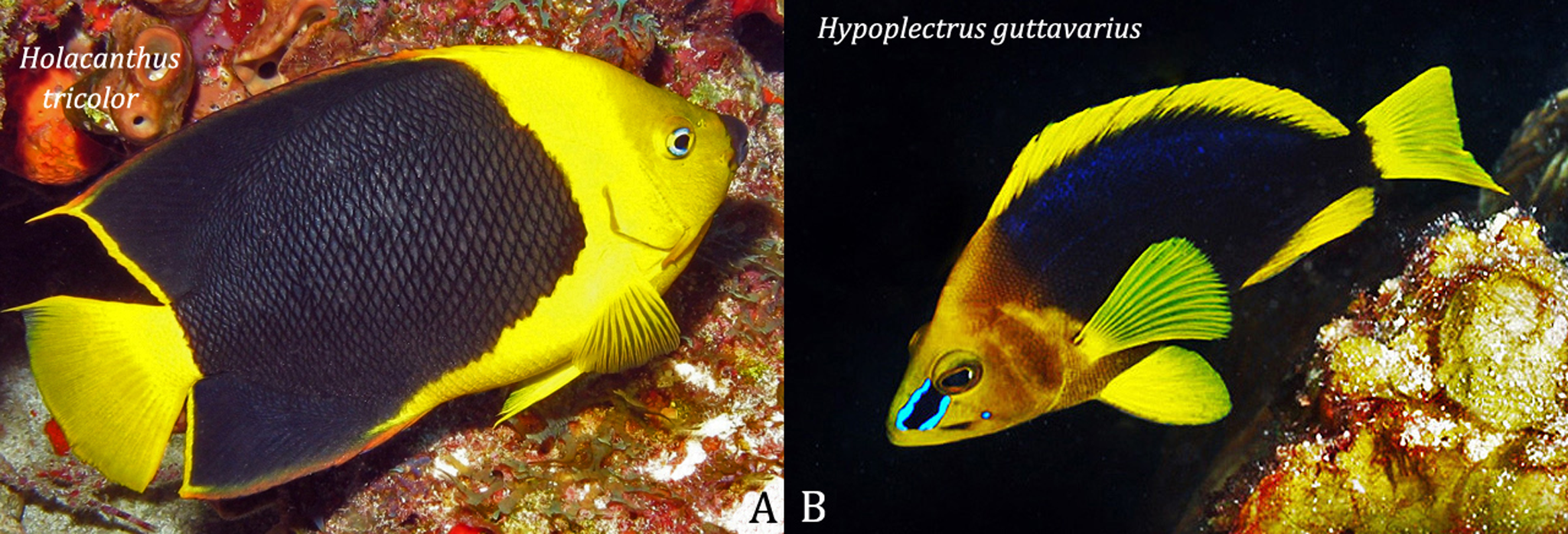

Supplement: Figure S4 — The shy hamlet Hypoplectrus guttavarius and its supposed model, the rock beauty angelfish Holacanthus tricolor . Photos: A - F Charpin; B – J Lyle. (TIF) [file pone.0054939.s004.tif]

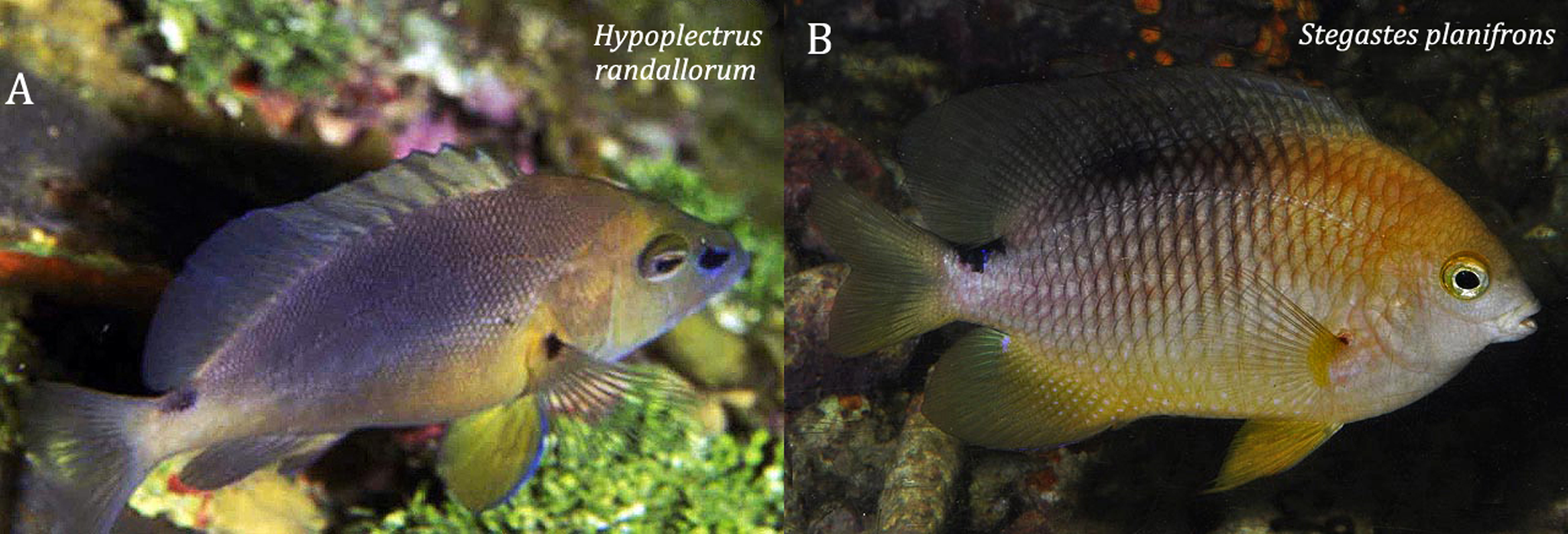

Supplement: Figure S5 — The tan hamlet Hypoplectrus randallorum and its supposed model, the threespot damselfish Stegastes planifrons. Photos: A - P Lobel; B - DR Robertson. (TIF) [file pone.0054939.s005.tif]
