# Supplementary material for: Who Resembles Whom? Mimetic and Coincidental Look-Alikes among Tropical Reef Fishes
Source: PLoS One. 2013 Jan 25;8(1):e54939. doi: 10.1371/journal.pone.0054939 (PMC3556028; doi:10.1371/journal.pone.0054939)
Supplement: Appendix S1 — General features of hamlet coloration, and five additional supposed mimetic hamlets. (DOC) [file pone.0054939.s006.doc]

**Appendix S1**

**General features of hamlet coloration**

Ground colors of hamlets contain varying tones of white, yellow, gold, orange, brown, black and blue, distributed in a variety of patterns on the head, body and fins. Superimposed on those ground colors are a number of discrete elements: (i) seven long dark vertical bars on the head and body; (ii) a large blue-ringed black blotch on the side of the snout; (iiii) a black saddle on the upper caudal peduncle; (iv) thin blue vertical lines on the head, and, sometimes, the body; (v) a black stripe along the upper and lower edges of the tail fin. In addition (vi) the pectoral fins may be clear, uniformly colored or bicolored, and may have a black spot at the upper fin base; (vii) in both dark and light colored species the eye and the tail, pectoral and pelvic fins may, singly or together, have the same or different colors to the body. These ground colors and pattern elements form a *Hypoplectrus* palette from which each hamlet species draws different combinations of items. All species (except perhaps *H. gemma*) exhibit variation in the intensity and extent and distribution of ground colors on the body and fins, and variation in the occurrence of different discrete elements [1]-[7] (and see [www.fishdb.co.uk](http://www.fishdb.co.uk/) , [www.reefguide.org](http://www.reefguide.org/)). All of the elements that make up the defining coloration of each species of “mimetic” hamlet can be found in one or more non-mimetic hamlets. The distinctive coloration of each species of hamlet, “mimetic” or otherwise, derives from a specific arrangement of existing elements, without any taxonomically unusual components in the coloration of “mimetic” species. The relationship between the coloration of the different species of hamlets can be thought of as a set of peaks of abundance (of varying width and height) in a multidimensional space of color hues and tones and pattern elements, with varying degrees of connection between peaks through valleys occupied by intermediately colored individuals. While the tips of the peaks that bear species names are recognizably distinct, the extent of variation in coloration and lack of clear associated genetic signals (see below) means that decisions as to what to name many individuals - species, hybrids, or variants of a named species - frequently are arbitrary.

Geographic variation in color patterns has been described in five common species [1], [3], [6], [8]. Although such color variants of a single species usually are not strictly allopatric, some are [4], [6]. In contrast to the great diversity of adult coloration, it appears that small juveniles of all (?) hamlet species have much the same coloration: a pale tan head and body, pale fins and two black spots (sometimes fused), each with a white spot immediately behind it, on the end of the caudal peduncle (e.g. [5], [9]). Thus, presently available information indicates that no “mimetic” hamlet’s small juvenile resembles the small juvenile of its putative “model” (see below).

Victor [7] recently described two new species of hamlets from the Gulf of Mexico and Florida peninsula that are genetically distinctly divergent from at least five of the remaining group of 14 named species, which occur mainly in the Caribbean. In the latter group, different hamlet species are delimited almost entirely on the basis of their distinctive coloration [1], [2], [10], 11]. There are only minor morphometric differences among many of the hamlets. Further, there is very little genetic differentiation among hamlet species within a locality [12]-[16], and only one is known to exhibit (limited) geographic genetic differentiation [16]. Laboratory experiments with four hamlet species show that two pairs are cross-fertile [3], [17]. Offspring of intraspecific matings of two species look like their parents, and interspecific matings of those two produce offspring that are intermediate in color to their parents [3]. Whether there are differences in the reproductive success of progeny of crosses between and within species is not known. Thus, although there is a genetic basis to “species” color differences, at present most “species” names other than those recently described by Victor [7] simply offer convenient labels for entities that do not readily fit conventional species concepts.

**Five additional “mimetic” hamlets**

***Hypoplectrus aberrans*:** In the yellow-belly hamlet the head and body typically are brown above and yellow below, and the fins (except for the brown spinous dorsal) are yellow. The relative amounts of yellow and brown on the head and body varies, some individuals have an iridescent blue cast on the brown back, there may be thin, bright blue vertical lines on the head, and some individuals have a dark blotch on the side of the snout (Figure S1, and see [5], [9]). Randall & Randall [10] noted a general similarity of *H. aberrans* to adults of two common herbivorous damselfishes, *Stegastes variabilis* and *S. planifrons*, and Thresher [1] proposed that *H. aberrans* mimics *S. variabilis* (see Figure S1). He briefly referred to parallel geographic variation in coloration of the two at the US Virgin Islands, where individuals of both have a blue back. Randall [18] noted that occasional individuals of *H. aberrans* in the Virgin islands have blue backs, and illustrated (his Figure 116) a fish from there with “typical” brown and yellow colors. The colors of individual *S. variabilis* vary considerably within a location, with differing amounts of blue, yellow and brown on different individuals of different sizes (DRR pers obs, and see [5], [9]). Thus the situation vis a vis coloration of *H. aberrans* and *S. variabilis* in the Virgin Islands, and elsewhere, requires clarification. Juvenile *S. variabilis* have quite different colors to those of their adults, and to those of *H. aberrans* (see [5], [9]). The only information available on the putative mimicry of *S. variabilis* by *H. aberrans* is on general similarities in coloration, form and size, general overlap in depth ranges, the fact that *H. aberrans* is less common than *S. variabilis*, and that the damselfish and hamlet have very different diets.

Thresher [1] also proposed that a blue-backed form of *H. aberrans* found in the central Caribbean mimics the similarly colored wrasse *Bodianus rufus,* which feeds on sessile benthic invertebrates [18]. *B. rufus* has a quite different body shape to *Hypoplectrus*, and reaches a much larger size than *H. aberrans* (lengths of 50 cm vs ~13 cm). Further, both wrasse and hamlet are relatively uncommon (compared to *Stegastes* spp), and *B. rufus* is also much more mobile than *Hypoplectrus*, which would reduce opportunities for any close behavioral association like that of *H. unicolor* with *Chaetodon capistratus*.

***Hypoplectrus chlorurus*:** The “typical” yellowtail hamlet is dark brown to blackish, with a bright yellow tail fin. Randall and Randall [10] noted the similarity of this color pattern to that of adults of the common herbivorous damselfish *Microspathodon chrysurus,* and Thresher [1] labelled *H. chlorurus* a mimic of *M. chrysurus* (see Figure S2). *M. chrysurus* is much more abundant than *H .chorurus*, and there is generalized overlap in their depth ranges and they are of roughly similar size and shape. However, *H chlorurus* varies in color: the tail fin can be clear rather than yellow, the pectorals and pelvics range from yellow-brown to yellow, the chest and belly may be suffused with yellow, and there may be prominent vertical blue lines on the head and front of the body [5], [6], [9]. Adults of *M. chrysurus* also have variable coloration: while some have dark brown bodies and yellow tails, others have much paler colors or even a uniform tan body and fins (DRR pers obs). All those features of coloration by both species reduce the resemblance of *H. chlorurus* to *M. chrysurus*. Small juveniles of *M chrysurus* are colored very differently to both their adults and of hamlets in general (see [5], [9]). Thus the resemblance of the color patterns of “model” and “mimic” is only generalized, and only related to part of the repertoire of colors exhibited by adults of the “model”. No further published information relating to a possible mimetic relationship is available.

***Hypoplectrus gemma*:** The blue hamlet has a plain iridescent blue body and fins, with a black stripe along the top and bottom edges of the tail fin. Similarities in their coloration led Randall & Randall [10] to propose that *H. gemma* is a mimic of the blue chromis, *Chromis cyanea* (see Figure S3). Randall & Randall [10] noted that *H. gemma* has a slightly more elongate body and slightly more concave tail than other hamlets, features that enhance its resemblance to *C. cyanea,* which has an elongated oval body and strongly concave tail fin*. C. cyanea* has the same general color pattern as *H .gemma*, but with the addition of a broad blackish stripe from the top of the snout along the upper body profile to the base of the tail fin. In addition, adult *C. cyanea* have ground color that can change between bright blue (like that of *H. gemma*) and dark bluey-purple. The coloration of juvenile *C. cyanea* is essentially the same as that of their adults (DRR pers obs), while the coloration of juvenile *H. gemma* has not been described. The coloration of adults of *H. gemma* (and *C. cyanea*) is relatively invariant compared to that of other “mimetic” hamlets and their “models”. Randall and Randall [10] noted that *H. gemma* is much less common than *C. cyanea*, that the two have very different diets (the blue chromis is a zooplanktivore), and that both occur in shallow water.

Fischer [2] suggested that, like *H. indigo, H. gemma* might prey on small *C .cyanea*. However, there are no field data relating to any relationship between these two species, and no published information on the diet of *H. gemma*. The issue of morphometric differences that might be linked to mimetic resemblance in this pair of species needs to be quantitatively revisited, as some photographs of *H. gemma* adults available on the internet show relatively deep bodies and a truncate tail fin, and small hamlets typically are more slender than adult conspecifics (DRR pers obs).

What was once considered to be a geographic color variant of *H. gemma* recently was named a separate species, *H. maya* [6]. *H. maya*, which lacks the black stripes on the tail fin seen in *H. gemma*, occurs in Belize, where *H. gemma* is unknown. *H. maya* is restricted to shallow habitats in the immense lagoon on the leeward side of the Belize barrier reef. Lobel [6] noted that *C. cyanea* is rare in that habitat and suggested that *H. maya’s* blue color may be cryptic in relation to a blue ascidian commonly found in mangrove habitat where *H. maya* also occurs. The few photographs available of adult *H. maya* show a fish with a form like that of adults of other *Hypoplectrus* spp – a relatively deep body with truncate tail (e.g. see [6]).

***Hypoplectrus guttavarius***: In the shy hamlet the upper three quarters of the head and body and the entire caudal peduncle are dark brown, the remainder of the fish is gold to orange, and there is a large, blue-ringed black blotch on each side of the snout. The upper-body brown area varies in tone and extent and sometimes is overlaid with a bright blue cast, and the snout blotch occasionally is missing. In addition there may be a dark tail-base saddle, and some fish have a dark bar through and under the eye [5], [9] (and see [www.fishdb.co.uk](http://www.fishdb.co.uk/) ). Thresher [1] proposed that *H. guttavarius* mimics the similarly colored angelfish *Holacanthus tricolor* (see Figure S4), which feeds on sessile benthic organisms. However, although those two species have very different diets, both are relatively uncommon, *H. tricolor* reaches a considerably larger size than the hamlet (30 cm vs ~15 cm), and has a broader depth range (down to 90 m vs to ~30m depth for *H. guttavarius*). Juveniles of *H. tricolor* have coloration very different from that of their adults [5], [9], and from that of any hamlet. No observations have been made on any interactions between these two species.

***Hypoplectrus randallorum***: The tan hamlet, *H. randallorum*, has a brown to yellowish brown body and fins, usually a large blue-ringed black blotch on each side of the snout, a small black spot on the upper pectoral base and, usually, a dark saddle at the top of the caudal peduncle [6]. The coloration of adult *H. randallorum* is similar to that of adults of the similarly sized damselfish, *Stegastes planifrons*, which Thresher [1] proposed is the model for a mimetic tan hamlet (see Figure S5). However, *S. planifrons* lacks any equivalent to the prominent snout blotches of *H. randallorum*, and large adults of *S. planifrons* often lose the black tail-base spot (DRR pers obs). Adult *S. planifrons* also lack other color elements seen in some adult *H. randallorum*, including blue marks on head, and black top and bottom tail borders. Juvenile *S. planifrons* have very different coloration to that of both conspecific adults and any hamlet [5], [9]. These various differences reduce the resemblance between hamlet and damselfish. *S. planifrons* is a very common damselfish, reaches about same size as *H. randallorum*, and feeds on benthic algae. Both species are commonest in shallow water. Thus there is a general resemblance of these two species, overlap in their habitat use, highly dissimilar diets, and the “model” far outnumbers the “mimic”. Nothing more is known about relations between these two species.

**References**

1. Thresher RE (1978) Polymorphism, mimicry, and the evolution of the hamlets (*Hypoplectrus*, Serranidae). Bull Mar Sci 28: 345–353.
2. Fischer EA (1980) Speciation in the hamlets (*Hypoplectrus:* Serranidae): a continuing enigma. Copeia 1980: 649–659.
3. Domeier ML (1994) Speciation in the serranid fish *Hypoplectrus*. Bull Mar Sci 54: 103–141.
4. Aguilar-Perera A (2004) Variations in Morphology and Coloration in the Black Hamlet, *Hypoplectrus nigricans* (Teleostei: Serranidae) CaribJ Sc 40:150-154.
5. Reefnet Inc (2007) Reef Fish ID: Florida, Caribbean, Bahamas. DVD 4.0. [www.reefnet.ca](http://www.reefnet.ca/).
6. Lobel PS (2011) A review of the Caribbean hamlets (Serranidae, *Hypoplectrus*) with description of two new species. Zootaxa 3096: 1–17.
7. Victor BC (2012) *Hypoplectrus floridae* n.sp. and *Hypoplectrus ecosur* n. sp., two new Barred Hamlets from the Gulf of Mexico (Pisces:Serranidae): more than 3% different in COI mtDNA sequence from the Caribbean *Hypoplectrus* species flock. J Ocean Sci Found 5: 1-19.
8. Aguilar-Perera A (2003) Abundance and distribution of hamlets (Teleostei: *Hypoplectrus*) in coral reefs off southwestern Puerto Rico: support for the multiple- species hypothesis. Carib J Sci 39:147-151.
9. Humann P (2008). Reef Fish Identification, Florida, Caribbean, Bahamas.3rd ed., Jacksonville, New World Publications, 481p.
10. Randall JE, Randall HA (1960) Examples of mimicry and protective resemblance in tropical marine fishes. Bull Mar Sci 10: 444–480.
11. Fischer EA (1980) The relationship between mating system and simultaneous hermaphroditism in the coral reef fish, *Hypoplectrus nigricans* (Serranidae). Anim Behav 28: 620–633.
12. Graves JE, Rosenblatt RH (1980) Genetic relationships of the color morphs of the serranid fish *Hypoplectrus unicolor*. Evolution 34: 240–245.
13. McCartney MA, Acevedo J, Heredia C, Rico C, Quenoville, B., Bermingham, E. & McMillan, O. 2003. Genetic mosaic in a marine species flock. Mol Ecol 12: 2963–2973.
14. Ramon ML, Lobel PS, Sorenson MD (2003) Lack of mitochondrial genetic structure in hamlets (*Hypoplectrus* spp.): recent speciation or ongoing hybridization? Mol Ecol 12: 2975–2980.
15. García-Machado E, Chevalier Monteagudo PP, Solignac M (2004) Lack of mtDNA differentiation among hamlets (*Hypoplectrus,* Serranidae) Mar Biol 144: 147–152.
16. Puebla O, Bermingham E, Guichard F, Whiteman E (2007) Colour pattern as a single trait driving speciation in *Hypoplectrus* coral reef fishes? Proc R Soc B 274:1265–1271.
17. Whiteman E A, Gage MJG (2007) No barriers to fertilization between sympatric colour morphs in the marine species flock *Hypoplectrus* (Serranidae). J Zool 272: 305–310.
18. Randall JE (1998) Caribbean reef fishes. 3^rd^ Ed. Neptune City, TFH, 368p.
